# An Unexpected Reaction between 5-Hydroxymethylfurfural and Imidazolium-Based Ionic Liquids at High Temperatures

**DOI:** 10.3390/molecules16108463

**Published:** 2011-10-11

**Authors:** Zehui Zhang, Wujun Liu, Haibo Xie, Zongbao K. Zhao

**Affiliations:** 1 Dalian Institute of Chemical Physics, Chinese Academy of Sciences, Dalian 116023, China; 2 Dalian National Laboratory for Clean Energy, Dalian 116023, China; 3 Key Laboratory of Catalysis and Materials Sciences of the State Ethnic Affairs Commission & Ministry of Education, College of Chemistry and Material Science, South-Central University for Nationalities, Wuhan 430074, China

**Keywords:** side reaction, HMF, ionic liquids, 1-butyl-2-(5’-methyl-2’-furoyl)imidazole

## Abstract

A new compound was detected during the production of 5-hydroxymethylfurfural (HMF) from glucose and cellulose in the ionic liquid 1-butyl-3-methylimidazolium chloride ([Bmim]Cl) at high temperatures. Further experiments found that it was derived from the reaction of HMF with [Bmim]Cl. The structure of new compound was established as 1-butyl-2-(5’-methyl-2’-furoyl)imidazole (BMI) based on nuclear magnetic resonance and mass spectrometry analysis, and a possible mechanism for its formation was proposed. Reactions of HMF with other imidazolium-based ionic liquids were performed to check the formation of BMI. Our results provided new insights in terms of side reactions between HMF and imidazolium-based ionic liquids, which should be valuable for designing better processes for the production of furans using biomass and related materials.

## 1. Introduction

Ionic liquids (ILs) are commonly composed of ions with combinations of organic cations and anions that exist as liquids at relatively low temperatures (<100 °C) [1,2,3]. Because up to 10^18^ compounds are expected to be potential ILs, the alias of “designer solvents” has been suggested [4]. Properties such as solubility, density, refractive index, and viscosity can be adjusted simply by making changes to the structures of the anion, the cation, or both [5,6,7]. ILs have attracted much interest as potentially greener alternatives to conventional organic solvents in various applications such as catalysis, separation science, polymer chemistry and so on [8,9,10,11]. In particular, ILs have been widely used in biomass conversion, thanks to the demonstration of solubilization of cellulose in ILs such as 1-butyl-3-methylimidazole chloride ([Bmim]Cl) [12,13].

5-Hydroxymethylfurfural (HMF) has been recognized as a versatile and key precursor for the production of fine chemicals, polymeric materials and biofuels [14,15,16,17]. In the past few years, production in ILs of HMF from hexoses, and even directly from cellulose and lignocellulosic materials, has been extensively studied by our group and others [18,19,20,21,22,23]. It is known that both the catalyst and the reaction environment have major effects in facilitating an efficient conversion of glucose into HMF [24]. Although HMF yields were generally higher in ILs than other solvent systems, attaining quantitative conversion was difficult. Since some cross-polymerizations during the course of HMF production, leading to the formation of colored soluble polymers and insoluble brown humic substances were documented, byproducts and low yields were routinely attributed to the cross-polymerization of HMF and/or its reaction intermediates [25,26].

Imidazolium-based ILs have been widely used due to their ready availability, low melting points and viscosity, and good stability to oxidative and reductive conditions [27]. They are considered to be “inert” solvents in most applications; however, it is well-established that the C2-position of the imidazolium ring can be de-protonated to form a stabilized carbene [28]. Some reactions involving ILs were reported in recent years. When imidazolium-based ILs were used as solvents in Baylis-Hillman reactions, base mediated deprotonation of the C2 position of the imidazolium moiety occurred, and the resulting nucleophile directly reacted with aldehydes [29]. Ebner *et al*. also showed that 1-alkyl-3-methylimidazolium could react with cellulose at its reducing end to forming a carbon-carbon bond [30]. This side reaction was strongly accelerated in the presence of base.

Recently, we have developed a microwave assisted production of HMF from glucose and cellulose in imidazolium-based Ils [31], and noticed that HMF yields were significantly lower upon excess microwave irradiation. During the course of purification of HMF from the reaction mixture by column chromatography on silica gel, we observed a new spot under UV light on the thin-layer chromatography slides. We isolated this compound and found the result interesting. In this paper, we would like to report the structure of this compound and propose a mechanism for its formation.

## 2. Results and Discussion 

### 2.1. Structural Analysis Based on One-Dimensional NMR Spectra

In our initial experiment, we tried to separate HMF from the reaction mixture by column chromatography on silica-gel after the dehydration reaction of glucose in [Bmim]Cl. A UV light-visible compound, eluted before HMF, albeit in small quantity. We suspected that it might be the result of a reaction between HMF and [Bmim]Cl. To test our speculation, we heated a mixture of HMF in [Bmim]Cl under the same conditions, and found that the UV light-sensitive compound was generated reproducibly.

**Figure 1:**
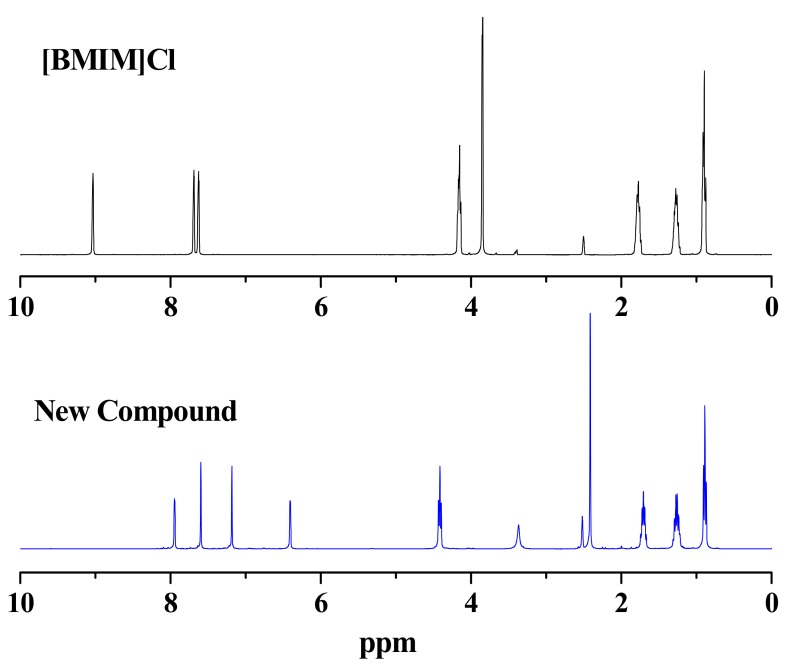
Comparison the ^1^H-NMR spectra of [Bmim]Cl and the new compound in DMSO-d_6_ at 30 °C.

We first measured the ^1^H-NMR of the new compound in DMSO-d_6_ (Figure 1). It was clear that the new compound had 16 hydrogen atoms and there were four low field hydrogen atoms likely associated with an aromatic ring.

Comparing the ^1^H-NMR spectrum of the new compound with that of [Bmim]Cl, it is easily to determine that the new -CH_2_-” (m, 1.74–1.67), “-CH_2_-” (m, 1.29–1.22), “CH_3_-” (t, 0.91–0.87), and “-CH_2_-” (t, 4.43–4.40) moieties were all fragments of a butyl group. A triplet “-CH_2_-” peak at δ 4.40–4.43 ppm indicated that this butyl group in the new compound was connected with a “heteroatom” or other strong electron-withdrawing group. In our reaction system, the butyl group connected with nitrogen in imidazolium ring was the most obvious option. In fact, the ^1^H-NMR chemical shifts of the butyl group in the new compound was almost the same with those in [Bmim]Cl, indicating that there is an imidazolium ring. A singlet peak at δ 2.41 ppm with three hydrogen atoms indicated that the compound contains a “-CH_3_” group. However, it was different from the methyl group in [Bmim]Cl, suggesting the “-CH_3_” group is unlikely connected with the nitrogen atom of the imidazolium ring. Moreover, the absence of the methyl group associated with the imidazolium ring suggested that the methyl group was cleaved from the imidazolium ring. It has been reported that [Bmim]Cl is unstable at high temperatures [32]. We also found that [Bmim]Cl could decompose into *N*-butylimidazole and methyl chloride at 250 °C. In addition, the NMR signals of the “-OH” and “-CHO” functions were missing in the spectra, suggesting that the side chains of the HMF structure were modified. Considering that [Bmim]Cl were known to react with aldehyde functionalities [29,30], our speculation was that the new compound was formed by the combination of HMF with the imidazolium ring through its C2 position.

**Figure 2:**
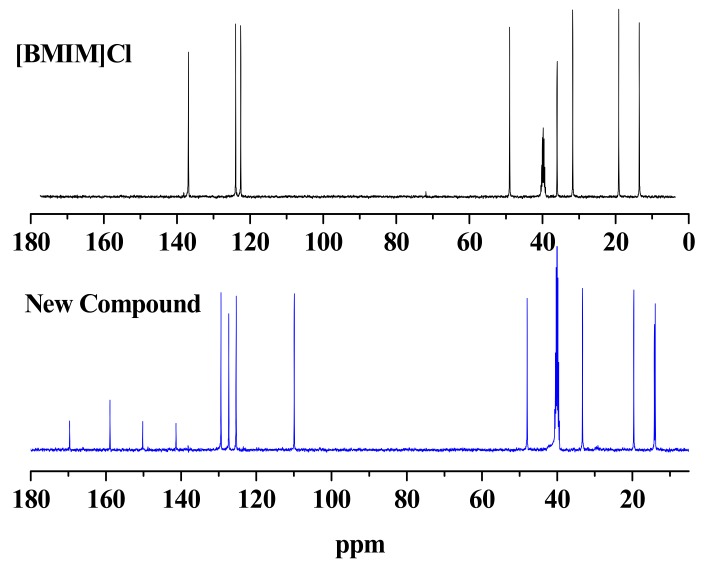
Comparison the ^13^C-NMR spectra of [Bmim]Cl and the new compound in DMSO-d_6_ at 30 °C.

The ^13^C-NMR spectrum of the new compound is shown in Figure 2. The chemical shift of the N-Me group on the imidazolium ring of [Bmim]Cl at δ 36.5 ppm and the chemical shifts of the carbons in HMF are δ 58.0 ppm (-CH_2_OH group) and δ 178.0 ppm (-CHO group) had all disappeared, which is consistent with the ^1^H-NMR results. A quaternary carbon shift at δ 169.0 ppm should be assigned as a ketone functionality, which could be derived from the aldehyde group of HMF. 

**Figure 3:**
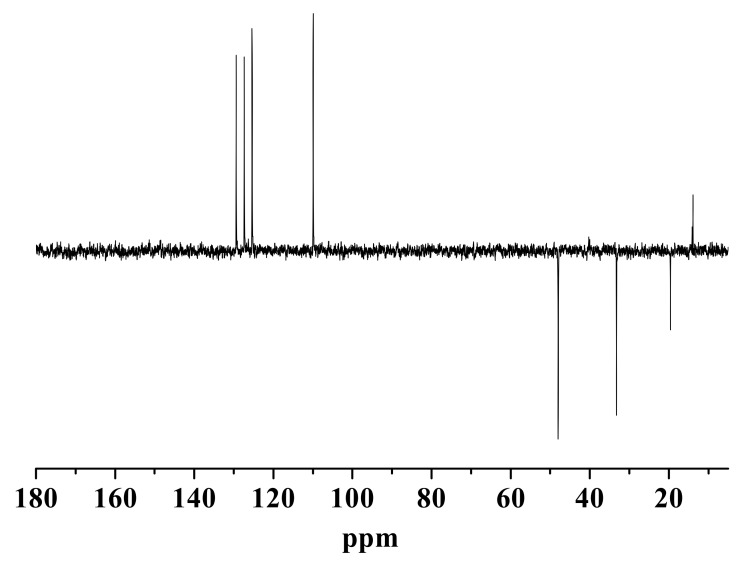
DEPT-135° NMR spectra of the new compound in DMSO-d_6_ at 30 °C.

**Figure 4:**
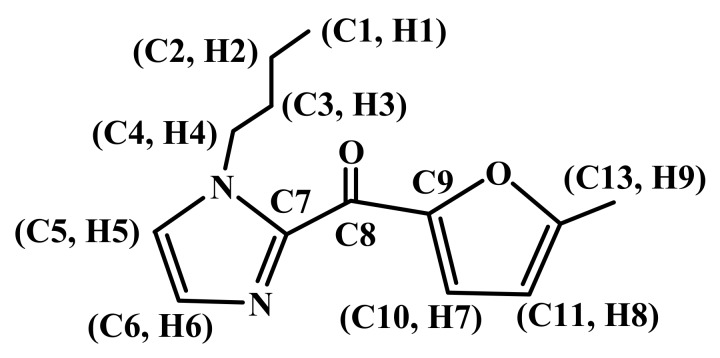
The proposed chemical structure of the compound BMI.

In addition, the DEPT-135° spectra of the new compound indicated there were seven low field unsaturated carbon atoms, namely four monosubstituted alkenyl carbons, and three disubstituted alkenyl carbons (Figure 3). As discussed above, the new compound posseses an N-butyl-substituted imidazole ring and a furan ring. Therefore, two disubstituted alkenyl carbons were easily associated with the furan ring structure of HMF origin, and four monosubstituted alkenyl carbons could be assigned to the imidazole and furan rings. The additional disubstituted alkenyl carbon might be associated with the C2 position of the imidazole ring, as a new carbon-carbon bond was likely formed to link the imidazole ring and the furan ring. Collectively, we could expect the following moieties/functionalities form the new compound, a ketone functionality, a methyl group not connected to a heteroatom, an N-butyl-substituted imidazole ring (likely with substitution at the C2 position), and a disubstituted furan ring. We thus proposed the new compound to be 1-butyl-2-(5’-methyl-2’-furoyl)imidazole (BMI), whose chemical structure is shown in Figure 4.

### 2.2. Further Structural Information of the Compound BMI

**Figure 5:**
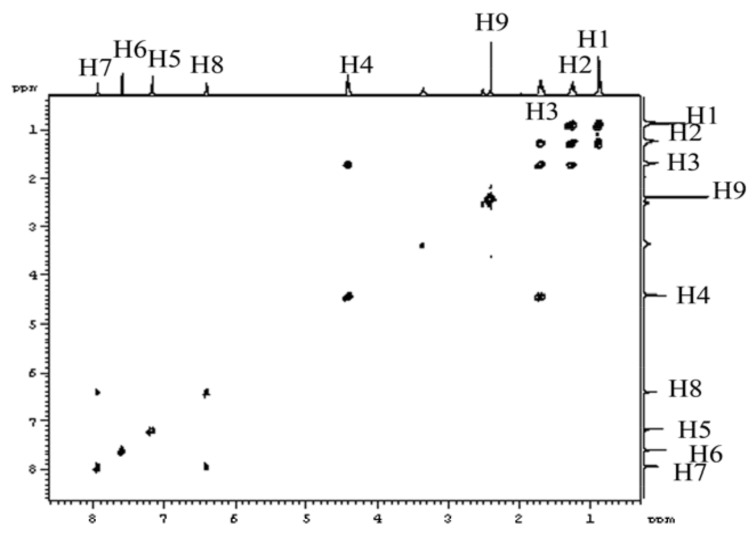
H-H COSY spectrum of the compound BMI in DMSO-d_6_ at 30 °C.

To gain further confirmatory structural information, we performed two dimensional NMR analysis. The H-H COSY spectrum of the sample is shown in Figure 5. It was easy to find that H1 (0.91–0.87, t, 3H) was coupled with H2 (1.29–1.22, t, 2H), H2 (1.29–1.22, m, 2H) was coupled with H3 (1.74–1.67, m, 2H), and H3 (1.74–1.67, m, 2H) was coupled with H4 (4.43–4.40, t, 2H). Therefore the presence of a butyl group was further confirmed. It also indicated that the methyl group H9 (2.41, s, 3H) connected with the furan ring does not couple with any hydrogen. In addition, two hydrogens in the furan ring H7 (7.95–7.94, d, 1H) and H8 (6.41–6.40, d, 1H) couple with each other.

**Figure 6:**
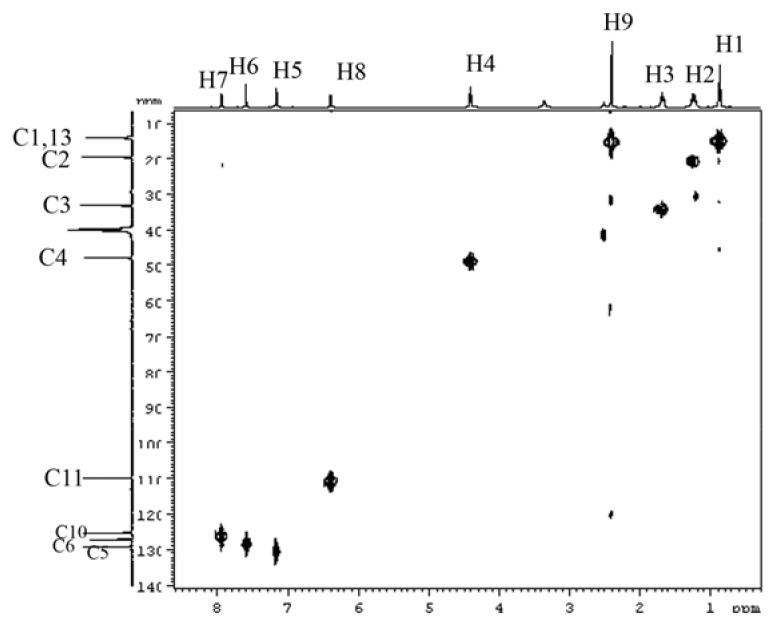
HMQC spectrum of the compound BMI in DMSO-d_6_ at 30 °C.

The HMQC spectrum of the sample (Figure 6) further established the connections between H and C atoms. That is, H_1_ (0.89, 3H) is connected with C1 (13.9), H2 (1.25, 2H) with C2 (19.6), H3 (1.70, 2H) with C3 (33.2), H9 (2.41, 3H) with C13 (14.1), H4 (4.41, 2H) with C4 (48.0), H8 (6.41, 1H) with C11 (109.9), H7 (7.95, 1H) with C10 (125.4), H5 (7.18, 1H) with C5 (127.3), and H6 (7.60, 1H) with C6 (129.4).

**Figure 7:**
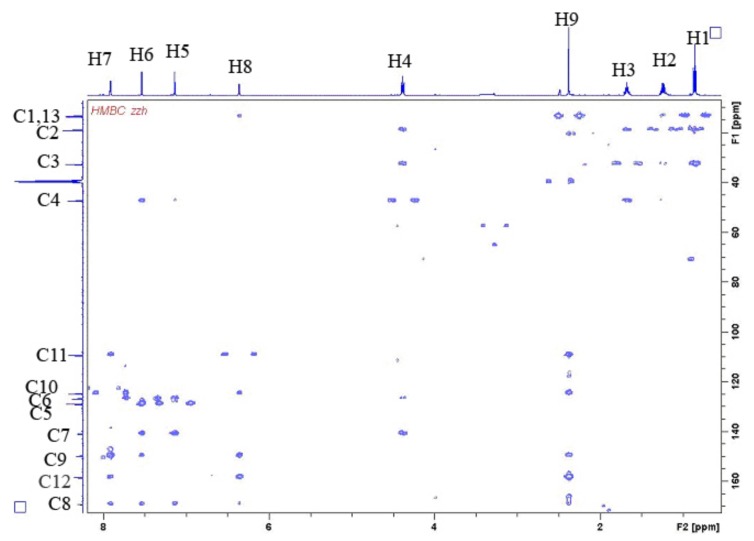
HMBC spectrum of the compound BMI in DMSO-d_6_ at 30 °C.

**Figure 8:**
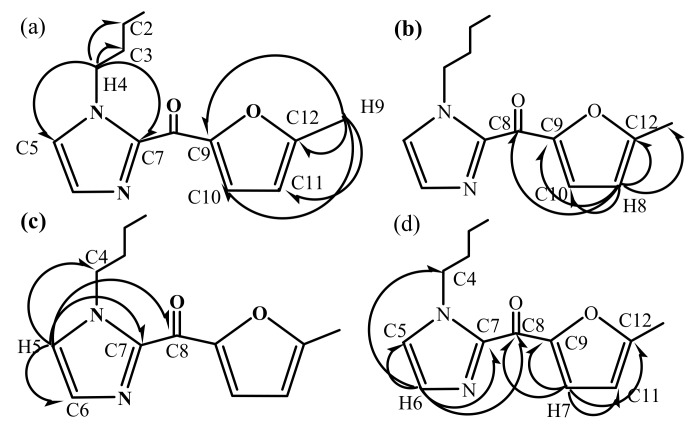
Schematic illustration of the HMBC results of compound BMI.

The structure of the new compound was further identified by heteronuclear multiple-bond correlation analysis. The HMBC spectrum is shown in Figure 7, and the bond connection information is shown in Figure 8. Briefly, we note in Figure 8a: H9 (2.41, 3H) coupling with C12 (158.9), C11 (109.9), C10 (125.4), C9 (150.2), H4 (4.41, 2H) with C2 (19.6), C3 (33.2), C5 (127.3) and C7 (141.4); in Figure 8b: H8 (6.41, 1H) coupling with C12 (158.9), C10 (125.4), C9 (150.2), C8 (169.6); in Figure 8c: H5 (7.18, 1H) coupling with C4 (48.0), C6 (129.4), C7 (141.4), C8 (169.6); in Figure 8d: H6 (7.60, 1H) coupling with C4 (48.0), C5 (127.3), C7 (141.4), C8 (169.6); and H7 (7.60, 1H) coupling with C9 (150.2), C8 (169.6), C11 (109.9), C12 (158.9).

**Figure 9:**
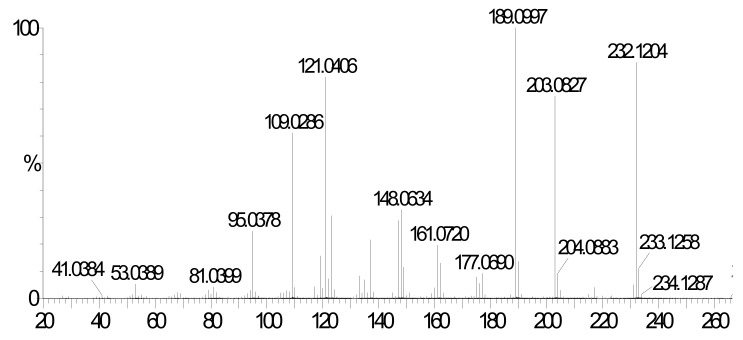
HRMS data of the compound BMI.

As indicated above, all two dimensional NMR spectra match well with the proposed chemical structure of BMI. Figure 9 shows the high resolution mass spectrum of BMI collected under electron impact ionization conditions. To match the exact mass of 232.1204 with 13 carbon atoms, the most probable molecular formula is C_13_H_16_N_2_O_2_. The compound BMI has an exact molecular formula of C_13_H_16_N_2_O_2_. The series of fragment ions at *m/z* 203.0827 and 189.0997 should result from the loss of an ethyl group [M-29]^+^ and a propyl group [M-43]^+^. The fragment ion at *m/z* 109.0286 should be attributed to the loss of an imidazolium ring moiety [M-123]^+^.

### 2.3. Possible Mechanism for the Formation of BMI

**Scheme 1:**
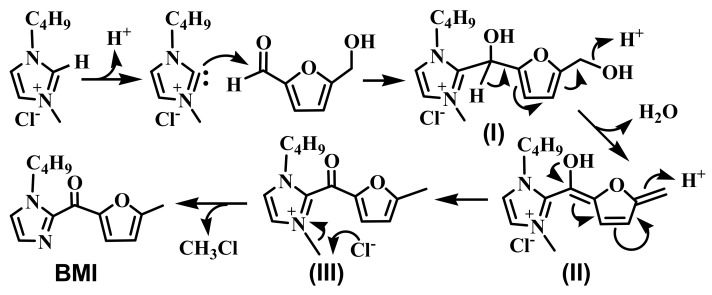
Proposed mechanism for the formation of the compound BMI.

The compound BMI can be envisioned as an adduct between HMF and [Bmim]Cl with loss of a H_2_O and a CH_3_Cl moiety. To propose a mechanism, it is very important to know the sequence of H_2_O release from the HMF side, CH_3_Cl release from the [Bmim]Cl side, and the C-C bond formation. As it has been demonstrated that [Bmim]Cl could expel a CH_3_Cl at high temperatures to give N-butylimidazole, we first checked the possibility of BMI formation involving N-butylimidazole. When N-butylimidazole and HMF were heated at 250 °C for 30 min, we could not detect any BMI by TLC. This observation suggested that N-butylimidazole is not directly involved, that is, CH_3_Cl release from the [Bmim]Cl side should not be the first event. The other possibility involves nucleophilic attack of the aldehyde group of HMF by the C2-deprotonated specie of [Bmim]Cl, leading to the formation of a C-C bond. Similar side reactions were known in imidazolium-based ILs [29]. Therefore, we proposed a mechanism as shown in Scheme 1. Briefly, the reaction starts with a nucleophilic attack of the aldehyde functionality of HMF to form the intermediate (I) featuring a carbon center substituted by a hydroxyl group and two heteroarmatic groups. Because the hydrogen atom attached to the newly formed carbon is acidic [33], the intermediate (I) released a H_2_O to give the enolate intermediate (II). Tautomerization of the conjugated enolate (II) afforded compound (III), which led to BMI upon discharging a CH_3_Cl via a nucleophilic attack by the chlorine ion.

### 2.4. Formation of BMI under Other Conditions

To provide more information about the formation of BMI, we heated a number of mixtures of ILs and HMF, and results are shown in Table 1. When HMF reacted with ILs composed of the cation 1-butyl-3-methyl-imidazolium and anions such as BF_6_^−^, NTf_2_^−^ and BF_4_^−^, or with [Emim]OAc, no BMI was detectable (Entries 2-5). According to the proposed mechanism, reactions may be trapped at their intermediates similar to compound (III) in Scheme 1, because PF_6_^−^, NTf_2_^−^, BF_4_^−^ and CH_3_CO_2_^−^ are less efficient than Cl^−^ in terms of nucleophilic attack on the methyl group attached to the quaternary nitrogen center. On the other hand, when HMF reacted with [Bmmim]Cl, there was also no BMI formation (Entry 6). This was likely because the C2 position of [Bmmim]Cl was blocked with a methyl group, and a nucleophile could not form. It should be noted that there was little HMF left in these cases, as formation of humic compounds via cross-polymerization reactions were indeed substantial [26]. Interestingly, when the reaction was heated at 200 °C, more BMI was recovered (Entry 7), but we could not obtain BMI at 180 °C, indicating that there was a step requiring high temperatures above 180 °C.

The observation of the compound BMI and the mechanistic insights described here are highly relevant to our current efforts to explore ILs as solvents in biomass conversion, particularly in the production of furans. The reactivity and stability of imidazolium-based ILs should be carefully scrutinized before the reactions were heated up to high temperatures. Although side reactions involving base mediated deprotonation of the C2 position of the imidazolium moiety were documented [29,30], this work indicated that the C2 position of the imidazolium moiety could also react with the carbonyl group in the absence of a base at high temperatures. Therefore, efforts should be devoted to designing more stable and inert ILs for biomass conversion.

## 3. Experimental Section

### 3.1. General

The ionic liquids 1-butyl-3-methylimidazolium tetrafluoroborate ([Bmim]BF_4_), 1-butyl-3-methyl-imidazolium bis(trifluoromethylsulfonyl)imide ([Bmim]NTf_2_) 1-butyl-3-methylimidazolium hexa-fluorophosphate ([Bmim]PF_6_), and 1-butyl-2,3-dimethylimidazolium chloride [Bmmim]Cl were synthesized according to the known procedures [34,35,36]. 1-Butyl-3-methylimidazolium chloride ([Bmim]Cl) and 1-ethyl-3-methylimidazolium chloride acetate ([Emim]OAc) were supplied by Lanzhou Greenchem ILS, LICP, CAS (Lanzhou, China). Glucose was purchased from ABCR GmbH & Co. (Karlsruhe, Germany). HMF was supplied by Beijing Chemicals Co., Ltd. (Beijing, China). N-Methylimidazole (99%) was obtained from Zhejiang Kaile Chemicals Co., Ltd. (Hangzhou, China). 1-Chlorobutane (98%) was obtained from Guangfu Fine Chemical Research Institute (Tianjin, China). Sodium tetrafluoroborate was purchased from Sinopharm Chemical Reagent Shanghai Co., Ltd. (Shanghai, China). Lithium bis(trifluoromethanesulfonyl)imide was purchased Acros Organics (Geel, Belgium). 1,2-Dimethylimidazole was purchased from Aladdin reagent Co., (Shanghai, China). DMSO-d_6_ was purchased from J&K Chemicals Co., Ltd. (Beijing, China). NMR spectra were measured in DMSO-d_6_ with a Bruker DRX-400 spectrometer (400.3 MHz for ^1^H-NMR and 100.6 MHz for ^13^C-NMR). Matrix-assisted laser desorption ionization time-of-flight mass spectroscopy (MALDI-TOF/MS) analyses were performed on a Micromass GC-TOF CA 156 MALDI-TOF/MS and operated in electron impact (EI) ionization mode.

### 3.2. Typical Procedure for Formation of the New Product 

In a typical run, HMF (100 mg) was added to [Bmim]Cl (2.0 g) and the mixture was heated at 250 °C for the desired time under atmospheric pressure with magnetic stirring, and then the reaction mixture was loaded on a silica gel column, eluted with petroleum ether and EtOAc (2:3, vol/vol). The new compound, about 17 mg (9.3%, based on HMF) was obtained, as yellowish oil (liquid at room temperature).

### 3.3. Characterization

Spectroscopic data for the new compound were as follows. ^1^H-NMR [(CD3)2SO] δ = 7.95–7.94 (d, *J* = 2.8 Hz, 1H), 7.60 (s, 1H), 7.18 (s, 1H), 6.41–6.40 (d, *J* = 2.8 Hz, 1H), 4.43–4.40 (t, *J* = 7.1 Hz, 2H), 2.41 (s, 3H), 1.74–1.67 (m, 2H), 1.29–1.22 (m, 2 H), 0.91–0.87 (t, *J* = 7.3 Hz, 3H). ^13^C-NMR [(CD3)2SO]: δ = 169.6, 158.9, 150.2, 141.4, 129.4, 127.3, 125.4, 109.9, 48.0, 33.2, 19.6, 14.1, 13.9. HRMS (EI) *m/z* found: 232.1204 (calculated for C_13_H_16_N_2_O_2_, M^+^. requires: 232.1212).

## 4. Conclusions

In conclusion, we have shown that HMF and [Bmim]Cl could react to form 1-butyl-2-(5’-methyl-2’-furoyl)imidazole at temperatures over 200 °C. Although it was minor impurity relative to the humic substances formed in these reactions, our mechanistic analysis indicated unambiguously that imidazolium-based ILs are not inert solvents for HMF production from hexoses. Our results provide new insights in terms of the production of HMF in imidazolium-based ILs, which should be valuable for designing better processes for the production of furans using biomass and related materials.

## Figures and Tables

**Table 1:** The results of HMF reaction with different ILs ^a^.

Entry	ILs	Temperature (°C)	Product mass (mg)	HMF recovery (%)
1	[Bmim]Cl	250	20	0
2	[Bmim]BF_4_	250	0	0
3	[Bmim]PF_6_	250	0	0
4	[Bmim]NTf_2_	250	0	0
5	[Emim]OAc	250	0	0
6	[Bmmim]Cl	250	0	0
7	[Bmim]Cl	200	28	0
8	[Bmim]Cl	180	0	0

^a^ Reaction conditions: a mixture of HMF (120 mg) in ILs (2.0 g) was heated for 25 min.
